# Effect of deoxyribonuclease I treatment for dementia in end-stage Alzheimer’s disease: a case report

**DOI:** 10.1186/s13256-016-0931-6

**Published:** 2016-05-28

**Authors:** Victor Tetz, George Tetz

**Affiliations:** 1 Human Microbiology Institute, Inc., 303 5th avenue, Suite 2012, New York, NY 10016 USA; 2 CLS Therapeutics, PO BOX 634, Bordeaux Court, Les Echelons, St Peter Port, Guernsey, Channel Islands GY1 3DR UK

**Keywords:** Alzheimer's disease, Neurodegeneration, Deoxyribonuclease I, Memantine, Dementia

## Abstract

**Background:**

Alzheimer's disease is the most common cause of dementia and is characterized by a progressive loss of brain tissue leading to amyloid-β accumulation and severe decline in cognitive function. The cause of Alzheimer’s disease is poorly understood, and available treatments are limited in their efficacy, particularly for patients with more severe symptoms.

**Case presentation:**

We report the case of a 77-year-old Caucasian man with severe dementia and behavioral disturbance secondary to Alzheimer’s disease treated with memantine who began adjunct treatment with deoxyribonuclease I. Prior to initiation of deoxyribonuclease I treatment, our patient appeared to be in a stuporous state, with a Mini-Mental State Examination score of 3 and a Functional Assessment Staging Test score of 7. After obtaining informed consent from family members, we started administration of 120 mg of deoxyribonuclease I per day (1500 KU/mg) for treatment of severe cognitive impairment.

**Conclusions:**

Our patient began to demonstrate rapid, considerable improvement in cognitive function 2 days following initiation of deoxyribonuclease I treatment. After 2 months of continued treatment, Mini-Mental State Examination and Functional Assessment Staging Test scores were 18 and 4, respectively.

## Background

Alzheimer's disease (AD) is an incurable, terminal illness characterized by progressive neurodegeneration and cognitive decline, accounting for roughly 60 to 80 percent of dementia cases [1]. More than 30 million people suffer from AD, and the number of patients is predicted to double every 20 years unless preventive measures are developed [2, 3]. The etiology of AD remains unclear; however, the characteristic accumulation of amyloid-β is likely associated with both genetic and environmental factors [4, 5].

Though several medications have been approved to treat the symptoms of Alzheimer’s (donepezil, rivastigmine, memantine, and galantamine), there is no cure for the underlying condition, and the current therapeutic strategies often provide merely temporary relief from symptoms and exhibit poor efficacy in patients with moderate to severe AD [6].

The present report details the case of a patient with severe end-stage AD who experienced significant symptomatic improvement upon treatment with deoxyribonuclease I (DNase I), an enzyme responsible for the cleavage of both human and microbial DNA, including cell-free DNA (cf-DNA). Therapeutic DNase I is a purified solution of recombinant human deoxyribonuclease I approved by the Food and Drug Administration (FDA) for the treatment of abnormal sputum viscosity in patients with cystic fibrosis [7]. As DNase I is an endonuclease, it has been suggested to have a beneficial role in the treatment of cancer; however the anticancer mechanism of action is not completely understood [8, 9].

## Case presentation

**Fig. 1:**
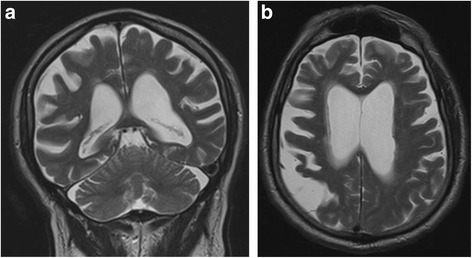
Atrophy and extensive gliosis of the left frontoparietal region in severe Alzheimer’s disease. Images (**a**) and (**b**) depict volume loss in end-stage Alzheimer’s disease with mild changes to the periventricular white matter. **a** Coronal T1-weighted magnetic resonance imaging scan showing marked progressive cortical atrophy of the parietal regions. **b** Transverse T1-weighted magnetic resonance imaging scan showing bilateral marked atrophy

A 77-year-old Caucasian man was diagnosed with dementia secondary to late-onset Alzheimer’s disease 30 months prior to his presentation at clinic, exhibiting behavioral disturbances, cognitive decline, and decreased ability to engage in activities of daily living. Approximately 14 months following the initial diagnosis, our patient began treatment with 10 mg of memantine per day [10, 11], though his cognitive condition continued to deteriorate, rapidly progressing to include such behavioral changes as aggressiveness and disinhibition, in addition to progressive amnesic syndrome, aphasia, bradykinesia, shuffling gait, loss of balance, and urinary incontinence. Further, our patient experienced a 20-pound weight loss, which is ordinarily indicative of poor prognosis in patients with AD [12, 13]. Analysis of cranial magnetic resonance imaging (MRI) scans revealed age-appropriate losses in volume and mild changes to the periventricular white matter (Fig. 1).

Thirteen months following initiation of memantine treatment, our patient’s total scores on the Mini-Mental State Examination (MMSE) and Functional Assessment Staging Test (FAST) were 10 and 5 points, respectively. He lost points on orientation to time and place, attention, memory, and visuospatial construction, and our patient was noticeably slower in completing the tasks.

He experienced additional difficulty in navigating turns and corners when walking, resulting in recurrent falls, and exhibited fluctuating levels of consciousness, alternating between periods of frank confusion and lucidity. However, he experienced no visual or auditory hallucinations.

A further 3 months later, and a total of 16 months following initiation of memantine treatment, our patient's experienced further deterioration of cognitive function. Our patient had fluctuating level of consciousness. His cognition fluctuated between periods of frank confusion and lucidity, however he had no visual or audial hallucinations.

He was unable to remember his name, the calendar date, day of the week, year, or place, and could not recognize family members. Additional impairments included slurred speech, expressive aphasia, loss of bowel/bladder control, and lack of coordination marked by an inability to sit, stand, or walk unassisted. Our patient became unresponsive to stimuli, with an MMSE score of 3 and a FAST score of 7. One month later, our patient’s relatives provided informed consent for treatment with 40 mg of human recombinant DNase I (1500 KU/mg) given orally three times a day in conjunction with his continued memantine therapy (10 mg daily). The DNase I was well tolerated, and no averse or unanticipated events were registered.

Our patient demonstrated considerable cognitive improvement beginning on the second day of DNase I treatment, becoming partially oriented to time and place, and once again recognizing and remembering the names of family members. He further became able to dress himself, including tying shoelaces and buttons, as well as walk independently, feed himself, and use an exercise bike. Neurologic abnormalities affecting his gait were significantly reduced. His MMSE score increased dramatically from 3 to 16, and his FAST score was reduced from 7 to 5. However, he continued to score low on the MMSE for measures of orientation to time and place, memory, and visuospatial construction.

Two months following the initiation of DNase I treatment (19 months following initiation of memantine treatment), our patient exhibited an MMSE score 18 and a FAST score of 4. Moderate improvements in memory were observed, although visuospatial construction continued to decline. He was better able to speak and interact with others, recognize relatives, and actively attend to television programs. Our patient further became able to perform calculations, play piano, chess, and walk independently.

## Conclusions

Treatment with DNase I in the present case allowed our patient to withdraw from a terminal state and resulted in significant improvements in cognitive and behavioral function, including the ability to walk and perform everyday tasks with near independence. DNase I was used as a repurposed FDA-approved medication [14]. Significant recovery was observed in all areas of cognitive and motor function, indicating the possibility of a DNase-sensitive target involved in generating the symptoms of AD.

Cell-free DNA, including bacteria-derived DNA, may be one such target [15]. Circulating cf-DNA has been observed to play an important role in the progression and maintenance of different disease states, including cancer, stroke, and other [16, 17]. Our previous research revealed its possible role as a therapeutic target in graft-versus-host diseases [18].

Though there are various suggestions regarding the origins of serum and plasma cf-DNA, many researchers have speculated that it results from apoptosis, necrosis, or both, or that this cf-DNA is secreted by cancer cells, microorganisms, hematopoietic cells, and/or neutrophils [19]. However, the physiological role of cf-DNA in the progression of these diseases remains to be discovered. Some have theorized that this role is mechanical in nature, increasing blood viscosity and leading to ischemia, while others suggest that cellular uptake of cf-DNA is involved in the development of metastatic conditions [20]. Little research has focused on the role of cf-DNA in neurodegenerative conditions, particularly AD. Further studies are required in order to evaluate the role of cf-DNA in the incidence and progression of neurodegenerative pathologies.

## Abbreviations

AD: Alzheimer's disease
cf-DNA: cell-free deoxyribonucleic acid
DNase I: deoxyribonuclease I
FAST: Functional Assessment Staging Test
MMSE: Mini-Mental State Examination
